# Long-duration head-down tilt bed rest confirms the relevance of the neutrophil to lymphocyte ratio and suggests coupling it with the platelet to lymphocyte ratio to monitor the immune health of astronauts

**DOI:** 10.3389/fimmu.2022.952928

**Published:** 2022-10-13

**Authors:** Pauline Jacob, Julie Bonnefoy, Stéphanie Ghislin, Jean-Pol Frippiat

**Affiliations:** Stress Immunity Pathogens Laboratory, UR 7300 SIMPA, Faculty of Medicine, Université de Lorraine, Vandœuvre-lès-Nancy, France

**Keywords:** head-down tilt bed rest, neutrophil/lymphocyte ratio (NLR), platelet/lymphocyte ratio (PLR), biomarker, spaceflight, inflammation, autoimmunity

## Abstract

The identification of safe and easily-determined-inflight biomarkers to monitor the immune system of astronauts is mandatory to ensure their well-being and the success of the missions. In this report, we evaluated the relevance of two biomarkers whose determination could be easily implemented in a spacecraft in the near future by using bedridden volunteers as a ground-based model of the microgravity of spaceflight. Our data confirm the relevance of the neutrophil to lymphocyte ratio (NLR) and suggest platelet to lymphocyte ratio (PLR) monitoring to assess long-lasting immune diseases. We recommend coupling these ratios to other biomarkers, such as the quantification of cytokines and viral load measurements, to efficiently detect immune dysfunction, determine when countermeasures should be applied to promote immune recovery, prevent the development of disease, and track responses to treatment.

## Introduction

Spaceflight is an extreme environment that threatens the health of astronauts. Indeed, numerous organ systems are altered by the space exposome, which comprises a multitude of unique stressors such as microgravity, radiation, isolation, confinement, disruption of sleep and circadian rhythms, high performance expectations, and risk of equipment failure or fatal accidents. Among affected biological systems, it is now well established that the space exposome induces immune system alterations that persist after return (1–3). These detrimental impacts on the immune system could compromise the defense against infections, toxins and cancer and consequently threaten the mission’s objectives. Indeed, it has been demonstrated that the immune system of approximately half of the astronauts who spent six months on the ISS is sensitive to spaceflight conditions (4).

Among immune disturbances that have been reported, several reports have indicated that spaceflight increases systemic inflammatory status. The analysis of plasma samples collected inflight in 28 astronauts participating in long-duration ISS missions revealed persistent low-grade systemic inflammation characterized by increased TNFα and IL-1RA plasma concentrations (5). This increase in proinflammatory cytokines was confirmed in 12 cosmonauts (6) and by a study of 14 previously uninvestigated cytokines in 13 astronauts participating in long-duration missions (7). Since persistent low-grade inflammation can lead to various diseases (8, 9), its monitoring appears crucial.

**Figure 1:**
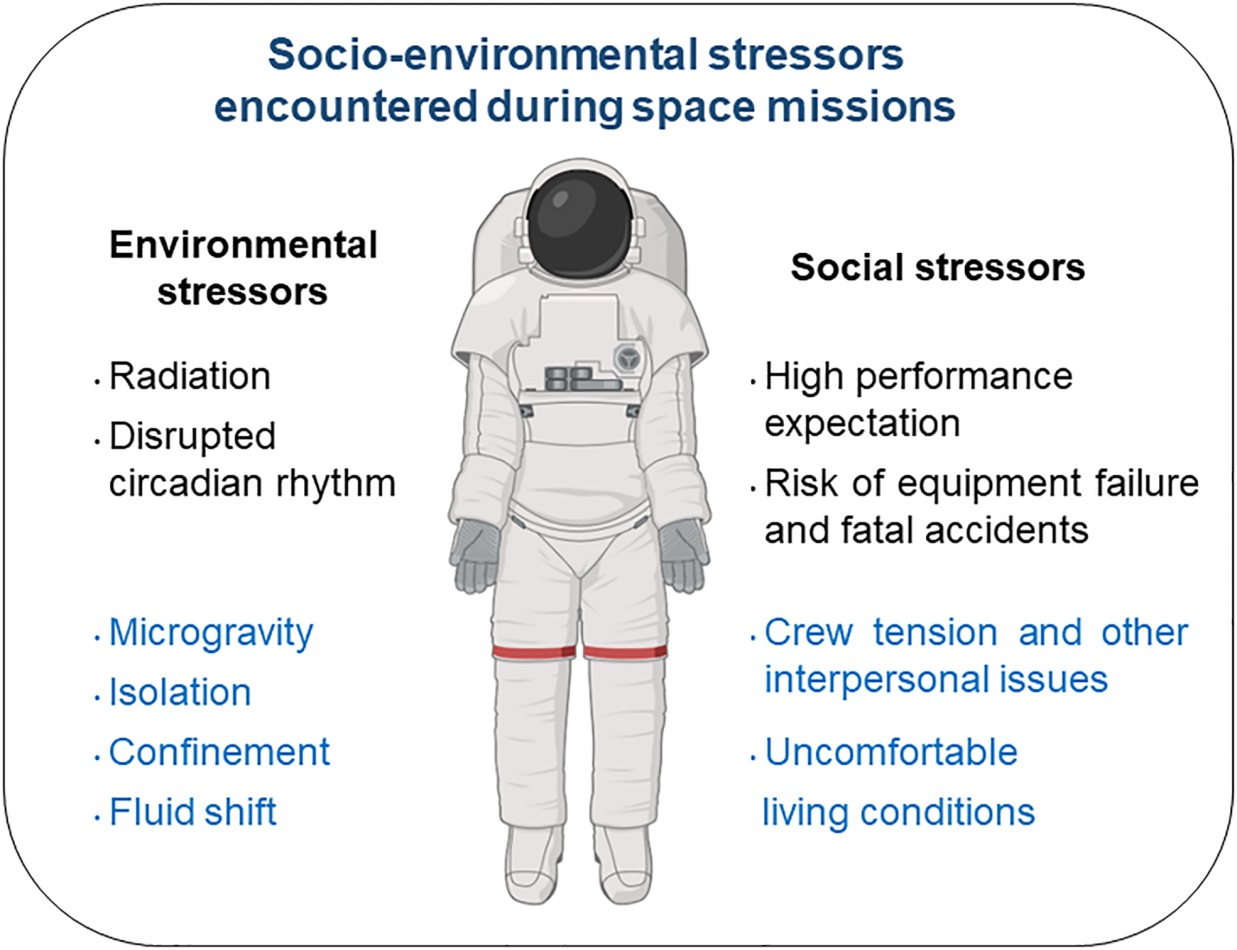
Socio-environmental stressors encountered during space missions. Those encountered during head-down tilt bed rest exposure appear in blue.

Given the limitations in the availability and the experimental protocols that can be performed with samples from astronauts, several ground-based models have been developed to mimic the effects of spaceflight. Among them, head-down tilt bed rest is the best and most integrated Earth-based analog of the microgravity of spaceflight (10) (see **Figure 1** presenting socio-environmental stressors present both during space missions and head-down tilt bed rest exposures). In a recent paper, Bonnefoy et al. (11) showed that, contrary to spaceflight, two months of head-down tilt bed rest seemed to lower the systemic inflammatory status in 20 healthy male volunteers. This hypothesis was supported by an increase in serum cortisone.

The neutrophil to lymphocyte (NLR) and platelet to lymphocyte (PLR) ratios are useful, inexpensive and readily available predictive biomarkers for several inflammatory, autoimmune, infectious and cardiovascular diseases and various types of cancer (12–17), all of which are potential complications associated with space missions (1, 2, 18, 19). Interestingly, a recent study suggested NLR as a biomarker for future inflight immune health monitoring (20). Consequently, we investigated in a group of 20 healthy male volunteers whether NLR and PLR are affected by 60 days of head-down tilt bed rest to confirm the relevance of these ratios as biomarkers of human immune system function during space missions. Indeed, such biomarkers, coupled with innovative technologies to monitor health and perform personalized diagnosis and medical intervention, are needed to ensure safe space exploration.

Our data confirm the relevance of NLR to monitor immune health during space missions and suggest that PLR monitoring could be used to assess long-lasting immune diseases.

## Materials and methods

### Subjects and ethics statement

Twenty healthy male volunteers (age: 34 ± 8; height: 176 ± 5 cm; weight: 73.5 ± 6.1 kg) were recruited for this exploratory longitudinal study. As indicated in Bonnefoy et al. (11), these subjects had no medical history or physical signs of disease. All were nonsmokers, healthy and not taking any drugs or medications. The head-down tilt bed rest protocol included 16 scientific protocols conducted in parallel and complied with the ethical standards of the 1964 Declaration of Helsinki. Protocols were approved (ClinicalTrial.gov database number NCT03594799) by the Institutional Review Board of the “Comité de Protection des Personnes Sud Ouest et Outre Mer I” (number ID RCB: 2016-A00401–50). All subjects gave their written informed consent before they started the study.

### Study design

This 2-month head-down tilt bed rest study, coordinated by the European and French National Space Agencies, was conducted at the Space Clinic of the Institute of Space Medicine and Physiology in Toulouse, France. It was originally designed to evaluate the efficiency of dietary supplementation with a cocktail of antioxidant substances. However, independent studies carried out on these subjects demonstrated that this supplementation had no effect on B-cell homeostasis (11), biomarkers of calcium homeostasis, bone formation and resorption (21), lumbar vertebral fat fraction (22), hemolysis and CO elimination (23), muscle deconditioning, oxidative damage, mitochondrial content and protein balance (24), neurobehavior (25), cardiac circadian rhythm (26) or adipose tissue immunometabolism (27). Therefore, individuals from the supplemented and non-supplemented groups could be pooled, allowing access to a group of 20 participants, which is quite a large group to have experienced long-duration head-down tilt bed rest.

From a practical point of view, this head-down tilt bed rest study was divided into two campaigns. In each campaign, 10 participants were randomly assigned to two groups in a double-blinded manner. Five of the participants were part of the non-supplemented group which did not receive the antioxidant cocktail during head-down tilt bed rest, whereas the five others were part of the supplemented group which received the antioxidant cocktail daily during head-down tilt bed rest. Details about the composition of the antioxidant cocktail as well as nutritional aspects monitored during this study can be found in Bonnefoy et al. (11). Each campaign consisted of a 2-month head-down tilt bed rest period (HDTBR) with a 14-day baseline data collection period (BDC) before HDTBR and a 14-day recovery (R) period after HDTBR. During HDTBR, subjects lay in a supine position with a -6° tilt to preserve simulated microgravity effects. All protocols and activities, including weighing and showering, were performed in a 6° head-down tilt position. The recovery period included a physical rehabilitation program tailored to each volunteer.

### NLR, GLR and PLR determination

Fasting venous blood was collected within 30 min after waking before other scientific protocols and several days after potentially traumatic procedures required by other participating teams (e.g., muscle biopsies). Neutrophils, granulocytes, platelets and lymphocytes were quantified four days before head-down tilt bed rest exposure (BDC-4), after 20, 49 and 60 days of head-down tilt bed rest (HDTBR20, HDTBR49 and HDTBR60, respectively), and 1, 13 and 60 days after the end of head-down tilt bed rest (R+1, R+13 and R+60, respectively) from blood samples collected in EDTA by the LaboSud Garonne laboratory (Accreditation 31 002 325 4, Labège, France) using SYSMEX XN9100 (SYSMEX, Roissy, France). NLR, GLR and PLR were calculated using neutrophil, granulocyte, platelet and lymphocyte levels in the complete blood count measurement.

### Statistics

NLR, GLR and PLR data were first analyzed for the statistical significance of the effects of dietary supplementation during head-down tilt bed rest with a repeated-measures two-way ANOVA with the Geisser-Greenhouse correction in combination with *post hoc* Sidak’s multiple comparison testing or, when data points were missing, with a Geisser-Greenhouse corrected linear mixed effects model using the restricted maximum likelihood method in combination with *post hoc* Sidak’s multiple comparison testing (GraphPad Prism 9.0). As no statistical significance was identified between supplemented and non-supplemented volunteers, pooled data for all volunteers per time point were analyzed for the statistical significance of the effects of head-down tilt bed rest and test days using a repeated-measures one-way ANOVA with the Geisser-Greenhouse correction in combination with *post hoc* Tukey multiple comparison testing or, when data points were missing, with a Geisser-Greenhouse corrected linear mixed effects model using the restricted maximum likelihood method in combination with *post hoc* Tukey multiple comparison testing (GraphPad Prism 9.0). No outliers were identified or removed. Information on age, height and weight was not considered because these parameters were not different between the supplemented and non-supplemented groups. An adjusted P value < 0.05 was considered statistically significant.

## Results

All participants completed the 60 days of head-down tilt bed rest, but three dropped out at R+60 during follow-up.

### NLR, GLR and PLR are not affected by dietary supplementation

**Figure 2:**
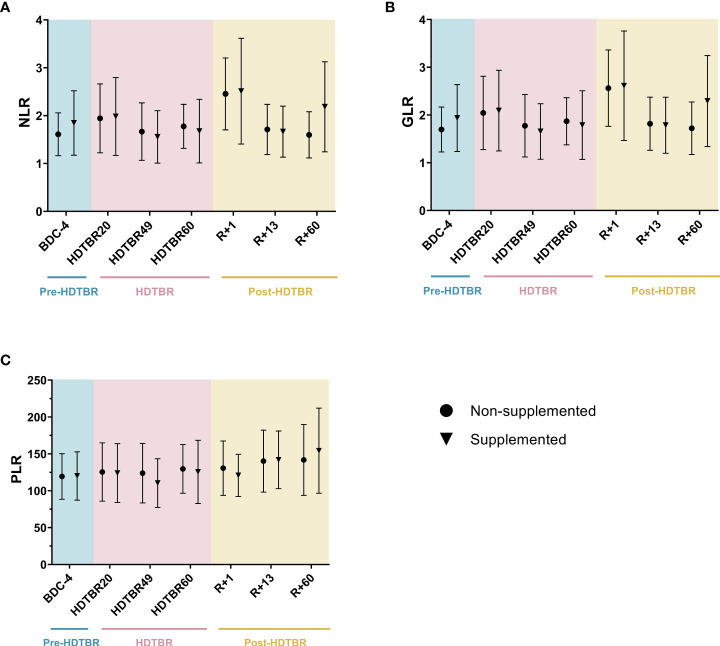
Comparison of NLR **(A)**, GLR **(B)** and PLR **(C)** ratios between the supplemented and non-supplemented groups at each time point of the head-down tilt bed rest study. Dots and triangles indicate the mean ± SD. n=10 in the supplemented group. n=10 in the non-supplemented group, except at R+60, where n=7. No statistically significant differences between the supplemented and non-supplemented groups were observed at any time point for any ratio using two-way ANOVAs, indicating that individuals of both groups could be pooled. BDC, baseline data collection (blue); HDTBR, head-down tilt bed rest (pink); R, recovery (yellow).

To confirm the non-effectiveness of the antioxidant supplementation used during this head-down tilt bed rest study, as mentioned above, we checked that NLR, GLR and PLR values were not significantly different between the supplemented and non-supplemented groups. **Figure 2** shows that none of these ratios were significantly different between the groups at each investigated time point. Thus, as expected, individuals from the supplemented and non-supplemented groups could be pooled, thereby giving us access to a large group having undergone long-duration head-down tilt bed rest.

### Effects of head-down tilt bed rest on NLR, GLR and PLR

**Figure 3:**
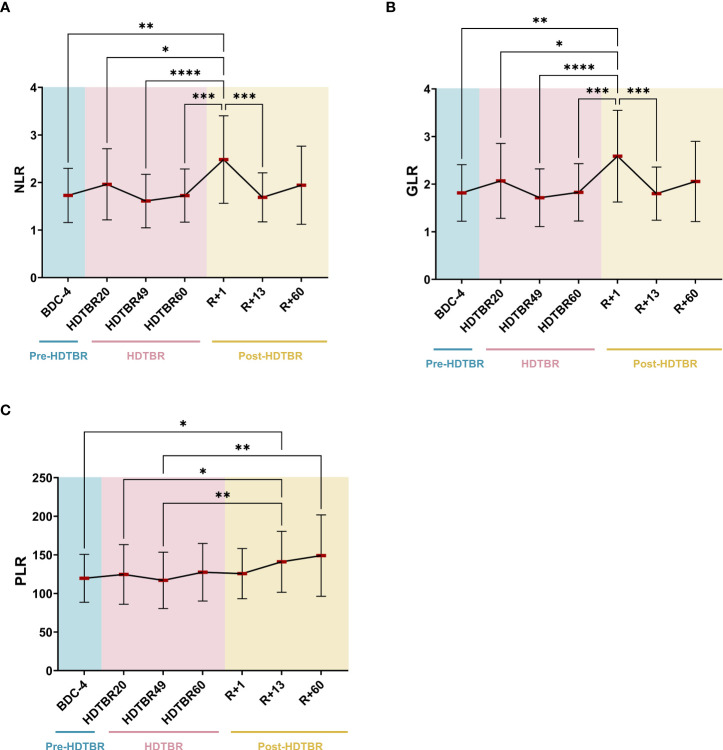
Evolution of NLR **(A)**, GLR **(B)** and PLR **(C)** ratios before, during and after two months of head-down tilt bed rest. As no statistically significant differences between the supplemented and non-supplemented groups were observed (**Figure 2**), individuals of both groups were pooled. n=20, except at R+60, where n=17. Horizontal bars indicate the mean. Statistically significant differences were revealed using either one-way ANOVA or a linear mixed effects model analysis. * p<0.05; ** p<0.01; *** p<0.001; **** p<0.0001. BDC, baseline data collection (blue); HDTBR, head-down tilt bed rest (pink); R, recovery (yellow).

Significant increases in NLR and GLR were noted at R+1. These ratios were increased by 50 ± 49% (mean ± SD) (p<0.01) and 48 ± 47% (p<0.01) at R+1 compared to BDC-4, respectively (**Figure 3**). Then, NLR and GLR ratios decreased at R+13 to reach a mean value similar to the one observed at BDC-4. The very similar evolution of NLR and GLR in this study occurs because basophil and eosinophil levels are low in peripheral blood in comparison to neutrophil levels, which represent 50-70% of the total leukocyte population. Regarding PLR, we noted that it gradually increased after head-down tilt bed rest. It increased by 20 ± 23% (p<0.05) at R+13 compared to BDC-4 and by 32 ± 22% (p<0.01) at R+60 compared to HDTBR49. Details about statistics can be found in **Supplementary Tables 1** and **2**.

## Discussion

Changes in the rate of circulating leukocytes serve as simple, rapid and economical markers of inflammation in many diseases (28). Such markers will be highly desirable for the early detection of immune changes during space missions to allow early diagnosis and timely treatment. Indeed, increasing and activating neutrophils will induce inflammation and the release of enzymes and reactive oxygen species that damage cells and tissues (29). There is evidence that platelets contribute to the inflammatory response (30), and lymphocyte counts may be affected by inflammation, infection and stress. In this context, NLR and PLR, which integrate two kinds of immune cells, are more reliable markers than the count of single immune cells.

### NLR and GLR are increased after head-down tilt bed rest

The only previous study assessing NLR and GLR as biomarkers to monitor immune status during space missions revealed that spaceflight significantly increased rodent NLR at the end of the 14-day Spacelab Life Sciences-2 mission and immediately postlanding (R+0). Then, rodent NLR decreased, suggesting a readaptation response to Earth’s gravity (20). This study also showed that the GLR of 23 astronauts who participated in 6-month ISS expeditions was elevated after 180 days in orbit and in samples collected within 2 to 3 hours postlanding (R+0). Postflight GLR later recovered to preflight baseline levels, again suggesting readaptation. Thus, this study revealed progressive increases in NLR and GLR that were statistically significant at the end of the space mission and immediately postlanding in rodents and humans, suggesting that these ratios may be useful biomarkers to monitor astronaut immune status. A very similar pattern was observed during this long-term head-down tilt bed rest. However, no increase in NLR and GLR was observed at HDTBR60, and a lower GLR increase was noted (mean GLR of 3.6 in astronauts at R+0 (20) versus mean GLR of 2.6 in head-down tilt bed rest volunteers at R+1). These differences are likely due to the fact that our head-down tilt bed rest was of a limited duration, that we did not collect blood at R+0, thereby allowing readaptation during 24 hours, and the absence of radiation which causes inflammation (31, 32). However, our results, which are deduced from 20 volunteers, are consistent with astronauts’ data, thereby confirming that NLR and GLR could potentially be useful markers to monitor the immune system during space missions. It is also very interesting to note that these slight increases in NLR and GLR are associated with major acute physical stress, landing in the case of spaceflight and the switch from 60 days of prone position to an upright position in the case of head-down tilt bed rest. These stresses certainly contribute to NLR and GLR peaks, as it is known that stress can lead to neutrophil demargination, and increases in NLR were observed up to 3 hours after intense training, with the extent of the increase depending on the training protocol (33). Thus, an increase in NLR or GLR seems to be a biomarker of acute physical stress, which can induce inflammation (34). In the future, it would be very interesting to study these markers before and after extravehicular activities, for example, to confirm their diagnostic interest.

### PLR increases after two months of head-down tilt bed rest

With regard to PLR, which has not been previously studied during space missions or head-down tilt bed rest exposure, we noted that this biomarker increased slightly and gradually after head-down tilt bed rest. This progressive increase could be due to a change in hematopoiesis. Indeed, Liu et al. (22) observed an enhancement of erythropoiesis during recovery in these volunteers, which was confirmed by Bonnefoy et al. (11). In addition, these authors noted an increase of platelets after HDTBR which they attributed to a slight increase in thrombopoiesis.

Interestingly, it has been reported that an increase in PLR correlates with disease activity of autoimmune diseases such as rheumatoid arthritis and systemic lupus erythematosus. Fu et al. (12) and Wu et al. (14) determined mean PLR values of 125 (min 67; max 212) and 99 (min 85; max 119) in healthy volunteers and of 179 (min 46; max 417) and 138 (min 94; max 223) in rheumatoid arthritis and systemic lupus erythematosus patients, respectively. Our mean PLR value at BDC-4 (120; min 71; max 189) is similar to that determined for healthy volunteers. Our mean PLR value at R+60 (149; min 93; max 284) is below the value of 179 for rheumatoid arthritis but close to the value of 138 determined for systemic lupus erythematosus.

Even if to date there is no clear evidence that spaceflight is associated with a risk of developing autoimmune disease, this possibility is an interesting avenue for further research as it is not quite comparable to the specific inflammation triggers observed in previous astronaut/cosmonaut subjects. Indeed, it was shown that murine medullary thymic epithelial cells (mTECs) are reduced after 14 days of hindlimb unloading (35), a model classically used to mimic the effects of spaceflight (36). Given that mTECs expressing tissue-specific antigens are critical for removing self-reactive T-cells and generating regulatory T-cells (37), this model may increase the risk of autoimmune disease. It was also shown that socioenvironmental stressors such as those encountered during space missions partially affect the murine TCRβ repertoire and could increase the self-reactivity of this repertoire (38). Postflight cytokine data collected from crew members revealed a decrease in TH1 cytokine expression (39), suggesting a potential TH2 cytokine shift that represents a significant clinical risk for TH2-related autoimmune diseases such as rheumatoid arthritis and systemic lupus erythematosus. Finally, our previous data revealed that two out of five analyzed cosmonauts presented significant changes in their IgM repertoire that persisted up to 30 days after landing (40). Thus, investigating PLR during space missions might be of interest because more prolonged-development diseases could occur (4).

### Limitations

This study is limited by the duration of head-down tilt bed rest exposure, interindividual differences which likely explain large variability in the data, and the fact that head-down tilt bed rest does not include a combination of some major spaceflight-encountered stressors such as radiation, disrupted circadian rhythm, high performance expectations, and the risk of equipment failure or fatal accidents. However, we are confident in our results because the subjects were under highly controlled study conditions and, as shown above, our data are consistent with previously collected space data.

## Conclusion and perspectives

In summary, this report highlights that simple, cost-effective, low-risk tests, such as determining neutrophil to lymphocyte (NLR) and platelet to lymphocyte (PLR) ratios, could aid in monitoring immune health during space missions. Our results are consistent with a previous study conducted on 23 astronauts who spent 6 months on the ISS (20), but further studies on larger cohorts will be needed to define space-appropriate NLR and PLR thresholds that can be used to predict risk. We recommend coupling these ratios to other biomarkers, such as cytokine quantification as increases in proinflammatory cytokines have been repeatedly observed in astronauts/cosmonauts (5–7), and viral load measurements as latent virus reactivation has also been frequently reported in these subjects and is a good biomarker of spaceflight-induced weakening of cell-mediated immunity (41). Such combination of biomarkers could allow to efficiently detect immune dysfunction, determine when countermeasures should be applied to promote immune recovery, prevent the development of disease, and track responses to treatment during space missions. Given recent progress in space biotechnology regarding the monitoring of peripheral white blood cells and differential cell counts (neutrophils, lymphocytes, monocytes, eosinophils, and basophils) within minutes from a fingerstick blood sample (42), the development of a Varicella zoster virus detection kit that can be used in space (43, 44), and the possibility of quantifying cytokines and stress markers using luminescence- or fluorescence-based assays (45), it can be hoped that such analyses will be implemented in the near future on the ISS and then on spacecrafts involved in future deep-space missions. These advances will also be of paramount importance to defeat diseases on Earth at the patient bed site, especially in medical deserts, and will contribute to telemedicine improvement.

## Data availability statement

The original contributions presented in the study are included in the article/**Supplementary Material**. Further inquiries can be directed to the corresponding author.

## Author contributions

PJ, JB and SG analyzed the data and performed statistical analyses. PJ and J-PF wrote the manuscript. JB and SG revised the manuscript. J-PF designed and supervised the project. J-PF obtained funding resources. All authors contributed to the article and approved the submitted version.

## Funding

This study was funded by the European Space Agency and the Centre National d’Etudes Spatiales (CNES, the French Space Agency). Investigators’ costs were covered by CNES (grants DAR 4800001108 and DAR 4800001163), the French Ministry of Higher Education and Research, the University of Lorraine, and the French State-Region Project Contract (CPER).

## Acknowledgments

We thank the staff of the Institute of Space Medicine and Physiology, Toulouse, France, for having organized and carried out this head-down tilt bed rest study, especially Dr. Marie-Pierre Bareille and Dr. Arnaud Beck. We also express our thanks to the volunteers for their participation and cooperation.

## Conflict of interest

The authors declare that the research was conducted in the absence of any commercial or financial relationships that could be construed as a potential conflict of interest.

## Publisher’s note

All claims expressed in this article are solely those of the authors and do not necessarily represent those of their affiliated organizations, or those of the publisher, the editors and the reviewers. Any product that may be evaluated in this article, or claim that may be made by its manufacturer, is not guaranteed or endorsed by the publisher.

## References

[1] Crucian BE Choukèr A Simpson RJ Mehta S Marshall G Smith SM. Immune system dysregulation during spaceflight: Potential countermeasures for deep space exploration missions. Front Immunol (2018) 9:1437. doi: 10.3389/fimmu.2018.01437 30018614 PMC6038331

[2] Akiyama T Horie K Hinoi E Hiraiwa M Kato A Maekawa Y. How does spaceflight affect the acquired immune system? NPJ Microgravity (2020) 6:14. doi: 10.1038/s41526-020-0104-1 32411817 PMC7206142

[3] Guéguinou N Huin-Schohn C Bascove M Bueb JL Tschirhart E Legrand-Frossi C. Could spaceflight-associated immune system weakening preclude the expansion of human presence beyond earth’s orbit? J Leukoc Biol (2009) 86:1027–38. doi: 10.1189/jlb.0309167 19690292

[4] Crucian B Babiak-Vazquez A Johnston S Pierson D Ott CM Sams C. Incidence of clinical symptoms during long-duration orbital spaceflight. Int J Gen Med (2016) 9:383–91. doi: 10.2147/IJGM.S114188 PMC5098747 27843335

[5] Crucian BE Zwart SR Mehta S Uchakin P Quiriarte HD Pierson D. Plasma cytokine concentrations indicate that *In vivo* hormonal regulation of immunity is altered during long-duration spaceflight. J Interferon Cytokine Res (2014) 34:778–86. doi: 10.1089/jir.2013.0129 PMC4186776 24702175

[6] Buchheim JI Matzel S Rykova M Vassilieva G Ponomarev S Nichiporuk I. Stress related shift toward inflammaging in cosmonauts after long-duration space flight. Front Physiol (2019) 10:85. doi: 10.3389/fphys.2019.00085 30873038 PMC6401618

[7] Krieger SS Zwart SR Mehta S Wu H Simpson RJ Smith SM. Alterations in saliva and plasma cytokine concentrations during long-duration spaceflight. Front Immunol (2021) 12:725748. doi: 10.3389/fimmu.2021.725748 34504500 PMC8422944

[8] Batista MA Calvo-Fortes F Silveira-Nunes G Camatta GC Speziali E Turroni S. Inflammaging in endemic areas for infectious diseases. Front Immunol (2020) 11:579972. doi: 10.3389/fimmu.2020.579972 33262758 PMC7688519

[9] Perdaens O van Pesch V. Molecular mechanisms of immunosenescene and inflammaging: Relevance to the immunopathogenesis and treatment of multiple sclerosis. Front Neurol (2022) 12:811518. doi: 10.3389/fneur.2021.811518 35281989 PMC8913495

[10] Pandiarajan M Hargens AR. Ground-based analogs for human spaceflight. Front Physiol (2020) 11:716. doi: 10.3389/fphys.2020.00716 32655420 PMC7324748

[11] Bonnefoy J Baselet B Moser D Ghislin S Miranda S Riant E. B-cell homeostasis is maintained during two months of head-down tilt bed rest with or without antioxidant supplementation. Front Immunol (2022) 13:830662. doi: 10.3389/fimmu.2022.830662 35251019 PMC8892569

[12] Fu H Qin B Hu Z Ma N Yang M Wei T. Neutrophil- and platelet-to-Lymphocyte ratios are correlated with disease activity in rheumatoid arthritis. Clin Lab (2015) 61:269–73. doi: 10.7754/clin.lab.2014.140927 25974992

[13] Kawamura Y Takeshita S Kanai T Yoshida Y Nonoyama S. The combined usefulness of the neutrophil-to-Lymphocyte and platelet-to-Lymphocyte ratios in predicting intravenous immunoglobulin resistance with Kawasaki disease. J Pediatr (2016) 178:281–284.e1. doi: 10.1016/j.jpeds.2016.07.035 27526622

[14] Wu Y Chen Y Yang X Chen L Yang Y. Neutrophil-to-Lymphocyte ratio (NLR) and platelet-to-Lymphocyte ratio (PLR) were associated with disease activity in patients with systemic lupus erythematosus. Int Immunopharmacol (2016) 36:94–9. doi: 10.1016/j.intimp.2016.04.006 27111516

[15] Demirdal T Sen P. The significance of neutrophil-lymphocyte ratio, platelet-lymphocyte ratio and lymphocyte-monocyte ratio in predicting peripheral arterial disease, peripheral neuropathy, osteomyelitis and amputation in diabetic foot infection. Diabetes Res Clin Pract (2018) 144:118–25. doi: 10.1016/j.diabres.2018.08.009 30176260

[16] Serban D Papanas N Dascalu AM Kempler P Raz I Rizvi AA. Significance of neutrophil to lymphocyte ratio (NLR) and platelet lymphocyte ratio (PLR) in diabetic foot ulcer and potential new therapeutic targets. Int J Low Extrem Wounds (2021) 18. doi: 10.1177/15347346211057742 34791913

[17] Isaac V Wu CY Huang CT Baune BT Tseng CL McLachlan CS. Elevated neutrophil to lymphocyte ratio predicts mortality in medical inpatients with multiple chronic conditions. Med (Baltimore) (2016) 95:e3832. doi: 10.1097/MD.0000000000003832 PMC4907663 27281085

[18] Delp MD Charvat JM Limoli CL Globus RK Ghosh P. Apollo Lunar astronauts show higher cardiovascular disease mortality: Possible deep space radiation effects on the vascular endothelium. Sci Rep (2016) 6:29901. doi: 10.1038/srep29901 27467019 PMC4964660

[19] Cortese F Klokov D Osipov A Stefaniak J Moskalev A Schastnaya J. Vive la radiorésistance!: converging research in radiobiology and biogerontology to enhance human radioresistance for deep space exploration and colonization. Oncotarget (2018) 9:14692–722. doi: 10.18632/oncotarget.24461 PMC5865701 29581875

[20] Paul AM Mhatre SD Cekanaviciute E Schreurs AS Tahimic CGT Globus RK. Neutrophil-to-Lymphocyte ratio: A biomarker to monitor the immune status of astronauts. Front Immunol (2020) 11:564950. doi: 10.3389/fimmu.2020.564950 33224136 PMC7667275

[21] Austermann K Baecker N Zwart SR Fimmers R Frippiat JP Stehle P. Antioxidant supplementation does not affect bone turnover markers during 60 days of 6° head-down tilt bed rest: Results from an exploratory randomized controlled trial. J Nutr (2021) 151:1527–38. doi: 10.1093/jn/nxab036 33831949

[22] Liu T Melkus G Ramsay T Sheikh A Laneuville O Trudel G. Bone marrow reconversion with reambulation: A prospective clinical trial. Invest Radiol (2021) 56:215–23. doi: 10.1097/RLI.0000000000000730 33038096

[23] Culliton K Louati H Laneuville O Ramsay T Trudel G. Six degrees head-down tilt bed rest caused low-grade hemolysis: A prospective randomized clinical trial. NPJ Microgravity (2021) 7:4. doi: 10.1038/s41526-021-00132-0 33589644 PMC7884785

[24] Arc-Chagnaud C Py G Fovet T Roumanille R Demangel R Pagano AF. Evaluation of an antioxidant and anti-inflammatory cocktail against human hypoactivity-induced skeletal muscle deconditioning. Front Physiol (2020) 11:71. doi: 10.3389/fphys.2020.00071 32116779 PMC7028694

[25] Brauns K Friedl-Werner A Gunga HC Stahn AC. Effects of two months of bed rest and antioxidant supplementation on attentional processing. Cortex (2021) 141:81–93. doi: 10.1016/j.cortex.2021.03.026 34044245

[26] Solbiati S Landreani F Turcato M Martin-Yebra A Costantini L Vaida P. Analysis of changes in cardiac circadian rhythms of RR and QT induced by a 60-day head-down bed rest with and without nutritional countermeasure. Eur J Appl Physiol (2020) 120:1699–710. doi: 10.1007/s00421-020-04404-7 32494859

[27] Trim WV Walhin JP Koumanov F Bouloumié A Lindsay MA Travers RL. The impact of long-term physical inactivity on adipose tissue immunometabolism. J Clin Endocrinol Metab (2022) 107:177–91. doi: 10.1210/clinem/dgab647 PMC8684473 34480570

[28] Summers C Rankin SM Condliffe AM Singh N Peters AM Chilvers ER. Neutrophil kinetics in health and disease. Trends Immunol (2010) 31:318–24. doi: 10.1016/j.it.2010.05.006 PMC2930213 20620114

[29] Nguyen GT Green ER Mecsas J. Neutrophils to the ROScue: Mechanisms of NADPH oxidase activation and bacterial resistance. Front Cell Infect Microbiol (2017) 7:373. doi: 10.3389/fcimb.2017.00373 28890882 PMC5574878

[30] Boilard E Nigrovic PA Larabee K Watts GFM Coblyn JS Weinblatt ME. Platelets amplify inflammation in arthritis *Via* collagen-dependent microparticle production. Science (2010) 327:580–3. doi: 10.1126/science.1181928 PMC2927861 20110505

[31] Parihar VK Allen BD Caressi C Kwok S Chu E Tran KK. Cosmic radiation exposure and persistent cognitive dysfunction. Sci Rep (2016) 6:34774. doi: 10.1038/srep34774 27721383 PMC5056393

[32] Barravecchia I De Cesari C Forcato M Scebba F Pyankova OV Bridger JM. Microgravity and space radiation inhibit autophagy in human capillary endothelial cells, through either opposite or synergistic effects on specific molecular pathways. Cell Mol Life Sci CMLS (2021) 79:28. doi: 10.1007/s00018-021-04025-z 34936031 PMC11072227

[33] Wahl P Mathes S Bloch W Zimmer P. Acute impact of recovery on the restoration of cellular immunological homeostasis. Int J Sports Med (2020) 41:12–20. doi: 10.1055/a-1015-0453 31747702

[34] Santos de Lima K Schuch FB Camponogara Righi N Chagas P Hemann Lamberti M Puntel GO. Effects of the combination of vitamins c and e supplementation on oxidative stress, inflammation, muscle soreness, and muscle strength following acute physical exercise: Meta-analyses of randomized controlled trials. Crit Rev Food Sci Nutr (2022) 9:1–14. doi: 10.1080/10408398.2022.2048290 35261309

[35] Horie K Kudo T Yoshinaga R Akiyama N Sasanuma H Kobayashi TJ. Long-term hindlimb unloading causes a preferential reduction of medullary thymic epithelial cells expressing autoimmune regulator (Aire). Biochem Biophys Res Commun (2018) 501:745–50. doi: 10.1016/j.bbrc.2018.05.060 29753741

[36] Globus RK Morey-Holton E. Hindlimb unloading: Rodent analog for microgravity. J Appl Physiol (2016) 120:1196–206. doi: 10.1152/japplphysiol.00997.2015 26869711

[37] Abramson J Anderson G. Thymic epithelial cells. Annu Rev Immunol (2017) 35:85–118. doi: 10.1146/annurev-immunol-051116-052320 28226225

[38] Fonte C Kaminski S Vanet A Lanfumey L Cohen-Salmon C Ghislin S. Socioenvironmental stressors encountered during spaceflight partially affect the murine TCR-β repertoire and increase its self-reactivity. FASEB J (2019) 33:896–908. doi: 10.1096/fj.201800969R 30052484

[39] Crucian BE Stowe RP Pierson DL Sams CF. Immune system dysregulation following short- vs long-duration spaceflight. Aviat Space Environ Med (2008) 79:835–43. doi: 10.3357/asem.2276.2008 18785351

[40] Buchheim J Ghislin S Ouzren N Albuisson E Vanet A Matzel S. Plasticity of the human IgM repertoire in response to long-term spaceflight. FASEB J (2020) 34:16144–62. doi: 10.1096/fj.202001403RR 33047384

[41] Kunz HE Makedonas G Mehta SK Tyring SK Vangipuram R Quiriarte H. Zoster patients on earth and astronauts in space share similar immunologic profiles. Life Sci Space Res (2020) 25:119–28. doi: 10.1016/j.lssr.2019.10.001 32414485

[42] Crucian B Valentine R Calaway K Miller R Rubins K Hopkins M. Spaceflight validation of technology for point-of-Care monitoring of peripheral blood WBC and differential in astronauts during space missions. Life Sci Space Res (2021) 31:29–33. doi: 10.1016/j.lssr.2021.07.003 34689947

[43] Cohrs RL. Rapid salive test for varicella zoster virus. Eur J Mol Clin Med (2015) 2:65. doi: 10.1016/j.nhtm.2014.11.036

[44] Mehta SK Pierson DL Ott CM. Early detection of immune changes prevents painful shingles in astronauts and in earth-bound patients. NASA Tech Innov (2010) 3:12–5. doi: 10.1055/a-1015-0453

[45] Frippiat JP. Space exploration and travel, future technologies for inflight monitoring and diagnostic. In: Shapshak P Balaji S Kangueane P Chiappelli F Somboonwit C Menezes LJ Sinnott JT, editors. Global virology III: Virology in the 21st century. Cham, Switzerland: Springer (2019). p. 471–84. doi: 10.1007/978-3-030-29022-1_16

