# Supplementary material for: Long-duration head-down tilt bed rest confirms the relevance of the neutrophil to lymphocyte ratio and suggests coupling it with the platelet to lymphocyte ratio to monitor the immune health of astronauts
Source: Front Immunol. 2022 Oct 13;13:952928. doi: 10.3389/fimmu.2022.952928 (PMC9606754; doi:10.3389/fimmu.2022.952928)
Supplement: Supplementary file 1 [file Table_1.docx]

**Supplementary Table 1.** F ratios, associated degrees of freedom and p-values, provided by GraphPad Prism, using a mixed-effect model because there were some missing values at R+60 (3 volunteers dropped out at this time point).

|  | **F ratio** | **Degrees of freedom** | **p-values** |
| --- | --- | --- | --- |
| **NLR** | **8.474** | **128** | **<0.0001** |
| **GLR** | **8.404** | **128** | **<0.0001** |
| **PLR** | **6.384** | **128** | **0.0005** |
