# Supplementary material for: Long-duration head-down tilt bed rest confirms the relevance of the neutrophil to lymphocyte ratio and suggests coupling it with the platelet to lymphocyte ratio to monitor the immune health of astronauts
Source: Front Immunol. 2022 Oct 13;13:952928. doi: 10.3389/fimmu.2022.952928 (PMC9606754; doi:10.3389/fimmu.2022.952928)
Supplement: Supplementary file 2 [file Table_2.docx]

**Supplementary Table 2.** Effect sizes and power for each analysis.

| **NLR** | | | ***Cohen’s d (effect size)*** | |  | | |
| --- | --- | --- | --- | --- | --- | --- | --- |
| ***Test details*** | ***Mean Difference*** | ***SE of difference*** | ***Value*** | ***Interpretation*** | ***Power*** | ***p-Value*** | ***Significance*** |
| BDC-4 vs. HDTBR20 | -0.2345 | 0.1381 | -1.70 | Very large | >99% | 0.625 | ns |
| BDC-4 vs. HDTBR49 | 0.1175 | 0.1256 | 0.94 | Large | 84% | 0.9616 | ns |
| BDC-4 vs. HDTBR60 | 0.0025 | 0.1096 | 0.02 | Very small | 3% | >0.9999 | ns |
| **BDC-4 vs. R+1** | **-0.7545** | **0.177** | **-4.26** | **Huge** | **>99%** | **0.0064** | ****** |
| BDC-4 vs. R+13 | 0.04 | 0.1088 | 0.37 | Small | 21% | 0.9997 | ns |
| BDC-4 vs. R+60 | -0.2155 | 0.2113 | -1.02 | Large | 87% | 0.9418 | ns |
| HDTBR20 vs. HDTBR49 | 0.352 | 0.1134 | 3.10 | Huge | >99% | 0.0714 | ns |
| HDTBR20 vs. HDTBR60 | 0.237 | 0.1205 | 1.97 | Very large | >99% | 0.4645 | ns |
| **HDTBR20 vs. R+1** | **-0.52** | **0.1575** | **-3.30** | **Huge** | **>99%** | **0.0483** | ***** |
| HDTBR20 vs. R+13 | 0.2745 | 0.1165 | 2.36 | Huge | >99% | 0.2694 | ns |
| HDTBR20 vs. R+60 | 0.01897 | 0.2159 | 0.09 | Very small | 4.50% | >0.9999 | ns |
| HDTBR49 vs. HDTBR60 | -0.115 | 0.05885 | -1.95 | Very large | >99% | 0.4721 | ns |
| **HDTBR49 vs. R+1** | **-0.872** | **0.1185** | **-7.36** | **Huge** | **>99%** | **<0.0001** | ******** |
| HDTBR49 vs. R+13 | -0.0775 | 0.08013 | -0.97 | Large | 86% | 0.9552 | ns |
| HDTBR49 vs. R+60 | -0.333 | 0.1748 | -1.91 | Very large | >99% | 0.5048 | ns |
| **HDTBR60 vs. R+1** | **-0.757** | **0.1289** | **-5.87** | **Huge** | **>99%** | **0.0002** | ******* |
| HDTBR60 vs. R+13 | 0.0375 | 0.07981 | 0.47 | Medium | 32% | 0.999 | ns |

| **GLR** | | | ***Cohen’s d (effect size)*** | |  | | |
| --- | --- | --- | --- | --- | --- | --- | --- |
| ***Test details*** | ***Mean Difference*** | ***SE of difference*** | ***Value*** | ***Interpretation*** | ***Power*** | ***p-Value*** | ***Significance*** |
| BDC-4 vs. HDTBR20 | -0.2525 | 0.1421 | -1.78 | Very large | >99% | 0.5776 | ns |
| BDC-4 vs. HDTBR49 | 0.1015 | 0.1298 | 0.78 | Large | 70% | 0.9841 | ns |
| BDC-4 vs. HDTBR60 | -0.012 | 0.1092 | -0.11 | Very small | 5% | >0.9999 | ns |
| **BDC-4 vs. R+1** | **-0.7715** | **0.1799** | **-4.29** | **Huge** | **>99%** | **0.006** | ****** |
| BDC-4 vs. R+13 | 0.015 | 0.1086 | 0.14 | Very small | 6% | >0.9999 | ns |
| BDC-4 vs. R+60 | -0.2411 | 0.2125 | -1.13 | Very large | 93% | 0.908 | ns |
| HDTBR20 vs. HDTBR49 | 0.354 | 0.1144 | 3.09 | Huge | >99% | 0.0727 | ns |
| HDTBR20 vs. HDTBR60 | 0.2405 | 0.1219 | 1.97 | Very large | >99% | 0.4616 | ns |
| **HDTBR20 vs. R+1** | **-0.519** | **0.157** | **-3.31** | **Huge** | **>99%** | **0.048** | ***** |
| HDTBR20 vs. R+13 | 0.2675 | 0.1186 | 2.26 | Huge | >99% | 0.3141 | ns |
| HDTBR20 vs. R+60 | 0.01144 | 0.2179 | 0.05 | Very small | 4.00% | >0.9999 | ns |
| HDTBR49 vs. HDTBR60 | -0.1135 | 0.06389 | -1.78 | Very large | >99% | 0.5778 | ns |
| **HDTBR49 vs. R+1** | **-0.873** | **0.1187** | **-7.35** | **Huge** | **>99%** | **<0.0001** | ******** |
| HDTBR49 vs. R+13 | -0.0865 | 0.08214 | -1.05 | Large | 91% | 0.9342 | ns |
| HDTBR49 vs. R+60 | -0.3426 | 0.1754 | -1.95 | Very large | >99% | 0.4773 | ns |
| **HDTBR60 vs. R+1** | **-0.7595** | **0.1299** | **-5.85** | **Huge** | **>99%** | **0.0002** | ******* |
| HDTBR60 vs. R+13 | 0.027 | 0.07952 | 0.34 | Small | 19% | 0.9998 | ns |

| **PLR** | | | ***Cohen’s d (effect size)*** | |  | | |
| --- | --- | --- | --- | --- | --- | --- | --- |
| ***Test details*** | ***Mean Difference*** | ***SE of difference*** | ***Value*** | ***Interpretation*** | ***Power*** | ***p-Value*** | ***Significance*** |
| BDC-4 vs. HDTBR20 | -5.006 | 4.724 | -1.06 | Large | 92% | 0.9323 | ns |
| BDC-4 vs. HDTBR49 | 2.576 | 6.222 | 0.41 | Medium | 26% | 0.9995 | ns |
| BDC-4 vs. HDTBR60 | -7.903 | 5.627 | -1.40 | Very large | >99% | 0.793 | ns |
| BDC-4 vs. R+1 | -6.025 | 6.785 | -0.89 | Large | 80% | 0.9701 | ns |
| **BDC-4 vs. R+13** | **-21.4** | **6.193** | **-3.46** | **Huge** | **>99%** | **0.0354** | ***** |
| BDC-4 vs. R+60 | -29.49 | 9.906 | -2.98 | Huge | >99% | 0.0996 | ns |
| HDTBR20 vs. HDTBR49 | 7.583 | 5.298 | 1.43 | Very large | >99% | 0.779 | ns |
| HDTBR20 vs. HDTBR60 | -2.897 | 4.871 | -0.59 | Medium | 47% | 0.9962 | ns |
| HDTBR20 vs. R+1 | -1.019 | 5.122 | -0.20 | Small | 9% | >0.9999 | ns |
| **HDTBR20 vs. R+13** | **-16.39** | **4.523** | **-3.62** | **Huge** | **>99%** | **0.0249** | ***** |
| HDTBR20 vs. R+60 | -24.48 | 8.378 | -2.92 | Huge | >99% | 0.1098 | ns |
| HDTBR49 vs. HDTBR60 | -10.48 | 4.76 | -2.20 | Huge | >99% | 0.3396 | ns |
| HDTBR49 vs. R+1 | -8.601 | 4.613 | -1.86 | Huge | >99% | 0.5247 | ns |
| **HDTBR49 vs. R+13** | **-23.98** | **5.637** | **-4.25** | **Huge** | **>99%** | **0.0065** | ****** |
| **HDTBR49 vs. R+60** | **-32.07** | **6.651** | **-4.82** | **Huge** | **>99%** | **0.0029** | ****** |
| HDTBR60 vs. R+1 | 1.878 | 4.909 | 0.38 | Small | 22% | 0.9997 | ns |
| HDTBR60 vs. R+13 | -13.5 | 5.053 | -2.67 | Huge | >99% | 0.1597 | ns |
